# Resveratrol and Pterostilbene Exhibit Anticancer Properties Involving the Downregulation of HPV Oncoprotein E6 in Cervical Cancer Cells

**DOI:** 10.3390/nu10020243

**Published:** 2018-02-21

**Authors:** Kaushiki Chatterjee, Dina AlSharif, Christina Mazza, Palwasha Syar, Mohamed Al Sharif, Jimmie E. Fata

**Affiliations:** 1 Doctoral Program in Biology, CUNY Graduate Center, New York, NY 10016, USA; kaushiki.chatterjee@csi.cuny.edu; 2 Department of Biology, College of Staten Island, New York, NY 10314, USA; dina95usa@aol.com (D.A.); Christinamazza19@gmail.com (C.M.); palwashasyar@yahoo.com (P.S.); Alsharif.mohamed@hotmail.com (M.A.S.)

**Keywords:** cervical cancer, resveratrol, pterostilbene, HPV E6, p53, cell cycle

## Abstract

Cervical cancer is one of the most common cancers in women living in developing countries. Due to a lack of affordable effective therapy, research into alternative anticancer compounds with low toxicity such as dietary polyphenols has continued. Our aim is to determine whether two structurally similar plant polyphenols, resveratrol and pterostilbene, exhibit anticancer and anti-HPV (Human papillomavirus) activity against cervical cancer cells. To determine anticancer activity, extensive in vitro analyses were performed. Anti-HPV activity, through measuring E6 protein levels, subsequent downstream p53 effects, and caspase-3 activation, were studied to understand a possible mechanism of action. Both polyphenols are effective agents in targeting cervical cancer cells, having low IC50 values in the µM range. They decrease clonogenic survival, reduce cell migration, arrest cells at the S-phase, and reduce the number of mitotic cells. These findings were significant, with pterostilbene often being more effective than resveratrol. Resveratrol and to a greater extent pterostilbene downregulates the HPV oncoprotein E6, induces caspase-3 activation, and upregulates p53 protein levels. Results point to a mechanism that may involve the downregulation of the HPV E6 oncoprotein, activation of apoptotic pathways, and re-establishment of functional p53 protein, with pterostilbene showing greater efficacy than resveratrol.

## 1. Introduction

Cervical cancer is one of the most prevalent cancers affecting women worldwide. It is the second most common cancer in developing countries and 11th in developed countries—these regional differences are often attributed to the lack of Pap smears, a preventative procedure often absent in underdeveloped areas [1,2]. It is widely accepted that the etiological factor that causes cervical cancer is chronic infection of the human papilloma virus (HPV), which is considered the most common sexually transmitted infection [3]. Every year about 500,000 women acquire the disease and 75% are from the developing countries [4]. Moreover, recent evidence indicates that HPV infection is on the rise in men, leading to higher incidences of penile and oropharyngeal cancer [5]. HPVs can be clinically classified as “low-risk” (LR-HPV) or “high-risk” (HR-HPV) depending on the relative tendency of the HPV lesions to transform into malignancy. HPV 16 and HPV 18 are the two most important cancer-causing, high-risk HPV [6]. HPV progression to cancer is dependent on prolonged infection by these high risk HPV viruses. The progression of HPV lesions to a neoplastic stage is dependent on several co-factors. Although there are approved HPV vaccines and drugs available, a problem is the affordability of these drugs in low income areas [7]. Two such vaccines are Cervarix^®^ and Gardasil^®^ [8], which renders prophylactic actions against cervical lesions associated with the most common oncogenic HPV types, 16/18, but effective therapeutic measures for post-infection lesions are currently not available. The major concern for the common chemotherapeutic medicines like cisplatin and paclitaxel are their adverse side effects [9,10]. The development of natural chemoprotective drugs that effectively target HPV infection could drastically reduce the incidence and progression of cervical cancer worldwide if they are not cost inhibitive and have low side effects. 

Of the varied groups of naturally occurring antioxidants, polyphenols have gained increased importance in cervical cancer since they have displayed potent antitumor properties in a number of cancers by targeting several pathways that are involved in cancer progression [11,12]. The current article uses cervical cancer cells to compare the tumor-inhibitory effects and mechanism of action of two such polyphenols, resveratrol and pterostilbene. Both resveratrol and pterostilbene are stilbenes, which is a class of natural polyphenolic compounds that have been studied for their anticarcinogenic activities. Resveratrol (3,4,5-trihydroxy-trans-stilbene) has been isolated from grapes, red wine, purple grape juice, peanuts, berries, and some medicinal plants [13]. Resveratrol is a widely studied stilbene compound having very low toxicity in the human system, and it is also known to modulate several pathways that are directly linked to cancer progression [14]. Both in vitro and in vivo cancer studies have shown resveratrol to inhibit cell proliferation and angiogenesis along with inducing pro-apoptotic properties [15]. The potential problem of using resveratrol as a chemoprotective agent is that it has low systemic bioavailability, which might lower its efficacy in the human system [16]. In order to overcome this, several efforts are being made to develop resveratrol derivatives with higher systemic bioavailability [17]. Pterostilbene (trans-3,5-dimethoxy-4-hydroxystilbene) is a naturally derived dimethylether analogue of resveratrol. Pterostilbene is believed to be produced in plants as a defense mechanism against some external microbial or fungal infection and is therefore considered a phytoalexin [18]. It has been isolated from grapevine leaves and blueberries [19]. Recently, pterostilbene has gained much attention as a possible anticancer agent, showing no toxicity in humans up to a dose of 250 mg/day [20]. 

Although the chemical structure of pterostilbene is closely related to resveratrol, the substitution of the hydroxyl group with a methoxy group in pterostilbene is believed to make the molecule more stable as well as increase its capacity to enter cells [21]. In addition, clinical studies have shown that the half-life and oral bioavailability of pterostilbene are significantly greater than those of resveratrol [20]. Studies on colon cancer cell lines have shown pterostilbene to be more potent than resveratrol in inhibiting DNA synthesis and in decreasing the expression of inflammatory genes responsible for cancer progression [22]. Although studies in other types of carcinomas show the potential efficacy of resveratrol and pterostilbene, there has been no study to the best of our knowledge that explores an anticancer mechanism that is specific for HPV-positive carcinoma. An in silico docking study has shown that resveratrol interacts with the p53 binding site of E6 residues [23]. E6 is a vital HPV oncoprotein essential for cervical cancer progression. E6 binds to tumor suppressor protein p53 and targets it for degradation by the ubiquitin proteasome pathway [24], thus causing uncontrolled cell proliferation. Here, we set out to compare the relative effectiveness of resveratrol against pterostilbene on cervical cancer cells, paying particular attention to their comparative IC_50_ values, changes in the levels of the HPV oncoprotein E6 and its target p53, as well as their comparative pro-apoptotic and anti-migratory capacities.

## 2. Materials and Methods 

### 2.1. Cell Culture 

Human cervical carcinoma HeLa cells were obtained from a commercial supplier (American Type Culture Collection, Manassas, VA, USA) and were cultured in Dulbecco’s Modified Eagle Medium: Nutrient Mixture F-12 (DMEM/F-12) (HyClone, GE Healthcare Life Sciences, Manassas, VA, USA), supplemented with 10% fetal calf serum (HyClone, GE Healthcare Life Sciences) and 0.1% Penicillin-Streptomycin Solution (HyClone). Cells were incubated in a 37 °C incubator with 5% CO_2_. 

### 2.2. Determination of IC_50_ Using WST-1 Assay

Seven thousand cells were plated on 96-well plates and allowed to grow for 24 h. Resveratrol (Acros, #430075000) or pterostilbene (TCI, #P1924) was serially diluted from 10–120 µM into DMEM/F-12 plus 1× insulin-transferrin-selenium (ITS) supplement (Invitrogen). Cells were treated with dilutions (in triplicate) for 24 h prior to performing a WST-1 (Water Soluble Tetrazolium salt-1) cell viability assay. The WST assay involved aspiration of the medium after treatment and rinsing three times with equal volumes of 1×Phosphate Buffered Saline (PBS), followed by the addition of 80 µL of 10% WST-1 (Clontech, Mountain View, CA, USA) in DMEM to each well. The plate was then incubated at 37 °C for 1 h and absorbance monitored at 440 nm using a plate reader. Results obtained were analyzed using GraphPad Prism 5 software to determine the IC_50_ using a standardized method [25,26].

### 2.3. Live Imaging

Images of untreated and treated (with resveratrol and pterostilbene) cells were taken every 10 min for 24 h to generate video files using a Zeiss Axio Observer Z1 microscope.

### 2.4. Clonogenic Assay

Two hundred thousand cells were plated on 6-well plates and allowed to grow for 24 h prior to treatment with pterostilbene (50 µM) and resveratrol (50 µM) for 24 h. After 24 h, cells were trypsinized to single cell suspensions. After cell counting, 150 viable cells from each treatment set were plated in one well from a 6-well plate and allowed to grow in complete DMEM/F-12 medium for 15 days. After said period of time, cells were washed once with 1× PBS then fixed and stained with 0.5% crystal violet in 6% glutaraldehyde for 30 min. The cells were briefly rinsed with tap water and allowed to air dry. Images of each well was taken and colonies were counted using ImageJ (NIH, Bethesda, Rockville, MD, USA). The plating efficiency and survival factor was calculated as determined previously [27].

### 2.5. Scratch Assay 

Twelve thousand cells were grown on 96-well plates until a confluent monolayer was formed. A scratch was made with a sterile p200 tip in each well through the center of the culture. The debris was washed off with serum-free media and a marking was made on the bottom of the plate to take images at the same location. Cells were then treated with different concentrations (5 µM and 20 µM) of resveratrol or pterostilbene and brightfield images were taken after 48 h to allow closure of the control scratch. The images were analyzed using ImageJ and the area of closure was measured according to previous published methods [28]. 

### 2.6. Flow Cytometry 

Two hundred and fifty thousand cells were cultured on 6-well plates and subsequently treated with resveratrol or pterostilbene (5 µM, 10 µM, and 15 µM) for 18 h. Cells were trypsinized, centrifuged, and washed with 0.1% Fetal Calf Serum (FCS) in 1× PBS solution and resuspended in 70% ethanol at −20 °C, which was added dropwise while shaking the samples vigorously. Fixed samples were kept at 4 °C for 1 h followed by washing twice in 1× PBS. Prior to flow cytometry, cells were incubated with RNase (500 µg/mL) for 30 min at 37 °C and then stained with propidium iodide (PI; 70 µM) for 30 mins. Cells were analyzed for DNA content by measuring PI fluorescence using an Accuri C6 flow cytometer (BD). 

### 2.7. Western Blot Analysis

Two hundred and fifty thousand cells were cultured on 6-well plates and subsequently treated with resveratrol (10 µM, 50 µM) or pterostilbene (10 µM, 50 µM) for a period of 22 h. Extraction of proteins from cultured cells was performed using M-PER Mammalian protein extraction reagent (Thermo Fisher, Waltham, MA, USA) with protease and phosphatase inhibitors. The total amount of protein in each well was quantified using the Lowry method. To resolve the proteins, 25 μg of protein was subjected to sodium dodecyl sulfate-polyacrylamide gel electrophoresis using a 10% acrylamide separating gel and then transferred to nitrocellulose membrane for 1 h. The membrane was blocked at room temperature for 1 h with 5% nonfat dry milk in Tris Tween Buffered Saline (TTBS). The nitrocellulose membrane was then incubated overnight with a p53 antibody (sc-6243) followed by incubation at room temperature for 1 h with anti-rabbit IgG conjugated with horseradish peroxidase. SuperSignal West Pico chemiluminescence substrate (Pierce) was used for detection following the manufacturer’s instructions. The membranes were scanned using APHA INNOTECH Fluorchem SP imaging system. Analysis of blots was done using ImageJ software.

### 2.8. Immunocytochemistry

Seven thousand HeLa cells were plated on 8-well chamber slides and allowed to grow for 48 h. Cells were then treated with different concentrations of resveratrol (5–50 µM) or pterostilbene (5–50 µM) for 22 h (for study of E6, p53 and cleaved caspase-3) and 18 h (for study of Phospho histone H3). All drug treatments were performed in serum-free DMEM/F-12 containing 1% supplement (ITS; insulin, transferrin, selenium; Gibco BRL, Grand Island, NY, USA). After treatment, cells were fixed in 4% paraformaldehyde at room temperature, rinsed with 1× PBS, and then permeabilized and blocked with 10% horse serum, 2% bovine serum albumin, and 0.5% Triton X-100 in PBS for 1 h. The cells were then incubated overnight with primary antibodies in blocking buffer. Subsequent to primary antibody treatment, the cells were washed and then incubated with the respective Fluorescein isothiocyanate (FITC) conjugated secondary antibodies for 3 h, followed by incubation with 4’,6-diamidino-2-phenylindole (DAPI) (10 µg/mL) and three washes with 1× PBS. The slides were then mounted with coverslips and cell images were acquired using a Zeiss Axio Observer Z1 microscope and an AxioVision 4.6.3-AP1. Images of different, randomly chosen fields were acquired with identical exposure times from each well for quantification. ImageJ was used to measure the fluorescence intensity and cell counting. The fluorescence intensities of E6 and P53 antibodies were normalized to DAPI intensity (blue). 

Antibodies Used: E6 antibody (sc-460, Santa Cruz Biotechnology, Dallas, TX, USA), p53 antibody (sc-6243), cleaved caspase-3 antibody (D175, 9661, CST), Phospho Histone H3 (Ser 10) antibody (06–570, Millipore, Burlington, MA, USA).

### 2.9. Statistical Analysis

Statistical analyses were performed using Microsoft Excel^®^ 2013 (Microsoft Corporation, Redmond, WA, USA) and GraphPad Prism^®^ 5 (GraphPad Software, Inc., La Jolla, CA, USA). Means and standard deviations were calculated for each group. One-way ANOVA with Tukey test was used to compare three or more datasets and determine the significance between the groups. ANOVA is a test of variance and post hoc Tukey test used is for the determination of significance between groups [29,30]. *p* < 0.05 was considered as significant.

## 3. Results

### 3.1. Pterostilbene Is More Potent in Eliminating HPV+ HeLa Cells Compared to Resveratrol

**Figure 1:**
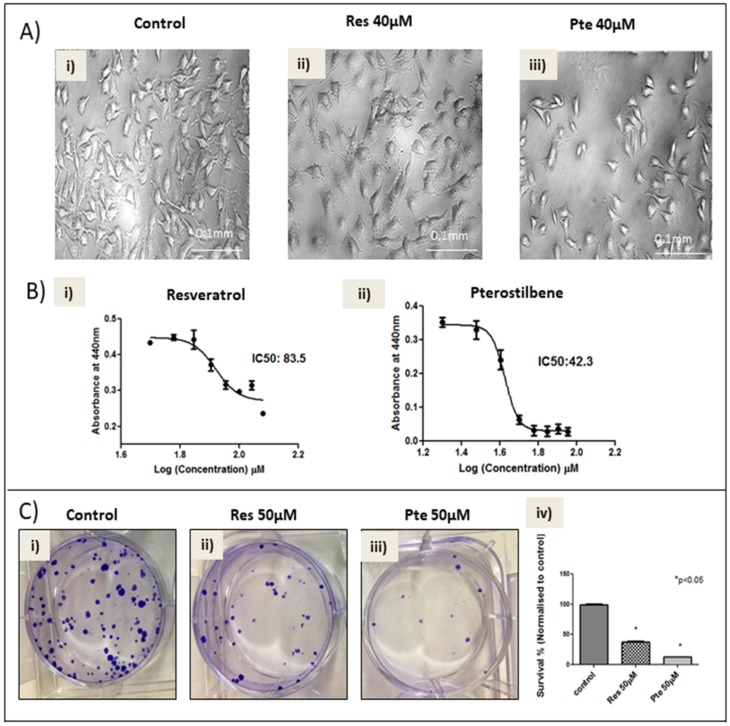
Pterostilbene is more potent in eliminating HeLa cervical cancer cells as compared to resveratrol: (**A**) Brightfield analysis of HeLa cells untreated (Ai) or treated for 24 h with 40 µM of resveratrol (Res; Aii) or 40 µM of pterostilbene (Pte; Aiii). Evidence of cell elimination was only seen robustly in cells treated with pterostilbene at 40 µM. (**B**) Analysis of IC_50_ values, generated by a Water Soluble Tetrazolium salt-1 (WST-1) assay after 24 h of exposure to resveratrol or pterostilbene indicates that pterostilbene (IC_50_ = 42.3 µM) is a more potent cytotoxic agent than resveratrol (IC_50_ = 83.5 µM; Bii). The graphs represent data from three independent experiments (mean ± S.E.M. (Standard error mean)). (**C**) Clonogenic assays performed to compare the relative effect of the two polyphenols on the clonogenicity of HeLa cells untreated (Ci) or treated with 50 µM of either resveratrol (Cii) or pterostilbene (Ciii). Results are from 15-days post-treatment and indicate that pterostilbene is more efficient in curbing the clonogenicity compared to resveratrol (Civ). Bar graph represents data from three independent experiments (mean ± S.E.M.; * *p* < 0.05; Civ).

In order to study the comparative cytotoxicity of pterostilbene and resveratrol on HeLa tumor cells, brightfield images (Figure 1A) and WST-1 cell viability assays (Figure 1B) were performed 24 h post-treatment. The brightfield images taken after 24 h of treatment (Figure 1A) showed that pterostilbene (40 µM) eliminates significantly more cells than resveratrol at the same concentration. Live imaging of cells treated with 60 µM of the two compounds show significantly more death and characteristic apoptotic blebbing in pterostilbene-treated cells when compared to untreated or resveratrol-treated cells (Supplementary Videos S1–S3). The WST-1 analysis revealed that although both pterostilbene and resveratrol eliminated HeLa cells significantly and in a dose-dependent manner, pterostilbene displayed a 1.97-fold lower IC_50_ when compared to resveratrol (42.3 µM vs. 83.5 µM; *p* < 0.05; Figure 1B). Additionally, both compounds, at 50 µM, significantly inhibited the clonogenicity of post-treated cells in a 15-day clonogenic assay (Figure 1C). Pterostilbene significantly reduced clonogenic survival by 87.5% compared to the control (*p* < 0.05), while resveratrol inhibited it by 63% (*p* < 0.05) (Figure 1C). Moreover, the difference between the survival percentages of the two treatment groups is significant (*p* < 0.05).

### 3.2. Inhibition of Cell Migration of HeLa Cells Treated with Pterostilbene and Resveratrol

**Figure 2:**
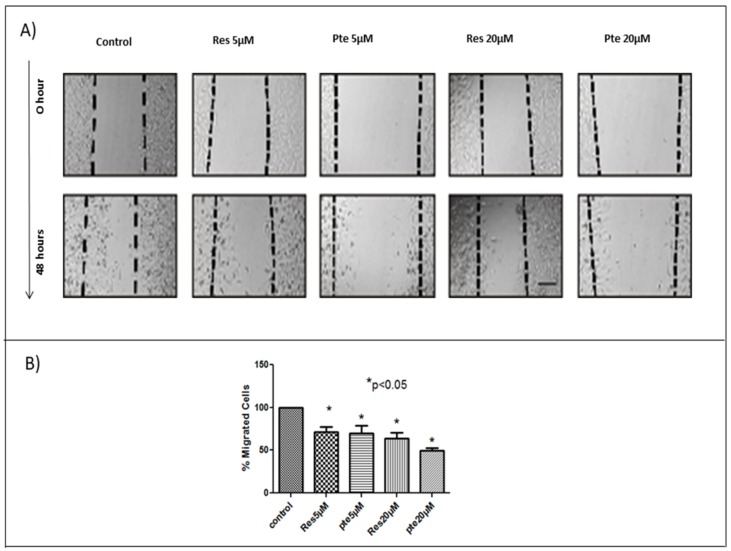
Resveratrol and pterostilbene inhibit cell migration: (**A**) HeLa cells were monitored for cell migration into a scratched “wound”. Cells were either untreated or treated with sub-lethal concentrations (5 µM and 20 µM) of resveratrol (Res) or pterostilbene (Pte). The extent of migration into the scratched area was calculated after 48 h and revealed that both resveratrol and pterostilbene significantly inhibit cell migration, although pterostilbene had greater anti-migratory effect. (**B**) The graphs represents data from triplicate sample experiments normalized to the control (mean % migrated cells ± S.E.M.; * *p* < 0.05). Scale bar: 0.05 µm.

To determine the comparative efficacy of resveratrol and pterostilbene in inhibiting HeLa cell migration, two different sub-lethal concentrations of each compound were used in a 48-h scratch assay (Figure 2). Based on the WST-1 results and brightfield images (unpublished), we found that cells treated with a concentration below 25 µM showed no signs of cellular toxicity. To avoid any cytotoxicity, we used lower concentrations of 5 µM and 20 µM. At sub-lethal concentrations of 5 µM and 20 µM, both resveratrol and pterostilbene significantly inhibited HeLa cell migration relative to untreated cells (*p* < 0.05; Figure 2). Pterostilbene was more effective in inhibiting HeLa cell migration at 20 µM when compared to resveratrol; however, this result was not significant and no differences were seen between the two compounds at 5 µM (Figure 2). In an effort to analyze the effects of resveratrol and pterostilbene on cell migration, we normalized the amount of migration into the scratch (wound) by untreated cells, to 100%. Relative to this control, resveratrol-treated cells migrated only 71.2% (5 µM) and 63.7% (20 µM), while cells treated with pterostilbene migrated only 69.5% (5 µM) and 49.2% (20 µM) (Figure 2).

### 3.3. Cell Cycle Arrest at S-Phase in HeLa Cells Treated with Low Concentrations of Resveratrol and Pterostilbene

**Figure 3:**
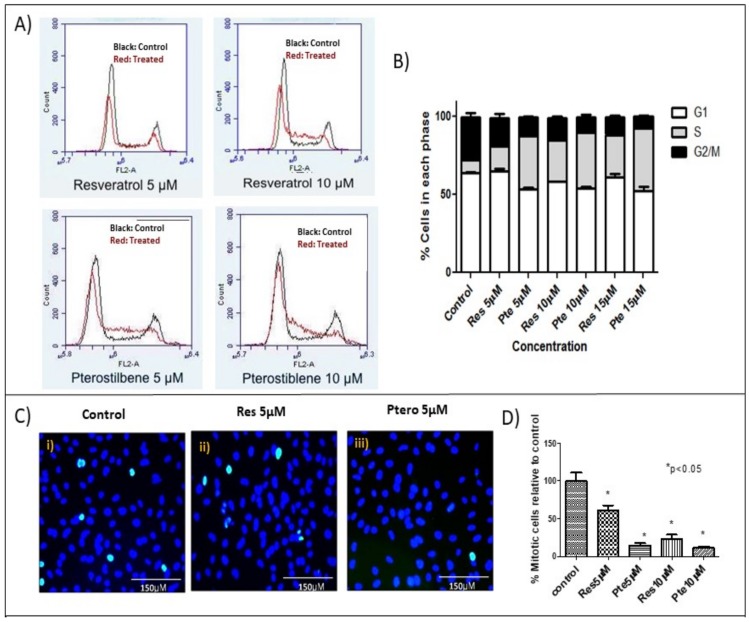
S-phase arrest in HeLa cells treated with low concentrations of resveratrol and pterostilbene: (**A**) Flow-cytometric evaluation of HeLa cells untreated or treated with sub-lethal doses of resveratrol (Res) and pterostilbene (Pte) for 18 h. Treated cells exhibited S-phase arrest and a subsequent decrease in the number of cells in G2/M. Pterostilbene was a more potent compound than resveratrol, showing a capacity to arrest cells at the S-phase at concentrations as low as 5 µM. (**B**) Graphical representation of the dose-dependent cell cycle effects induced by resveratrol and pterostilbene at three different concentrations (5 µM, 10 µM, and 15 µM). (**B**) The graph represents data from triplicate sample experiments normalized to the control (mean % cells in each phase ± S.E.M.) (**C**) Immunofluorescent images of HeLa cells probed for the M-phase marker phospho-histone-H3 (serine10). HeLa cells were untreated or treated with 5 µM and 10 µM of resveratrol or pterostilbene. Immunofluorescent images display a decrease of histone-H3 in cells treated with both the compounds, the effects at 5 µM of pterostilbene is much greater than those of resveratrol (at 5 µM). (**D**) Graphical representation of the percent of mitotic cells calculated from immunofluorescent images reveal that resveratrol and to a greater extent pterostilbene are effective in decreasing the number of mitotic HeLa cells. The graph represents data from experiments obtained from triplicate samples normalized to the control (mean % mitotic cells ± S.E.M.;* *p* < 0.05).

In order to compare the effect of sub-lethal doses of either resveratrol or pterostilbene on the cell cycle of HeLa cells, treatment was carried out with three different concentrations (5 µM, 10 µM, and 15 µM) of the two compounds for 18 h prior to flow cytometric analysis (Figure 3A). Flow cytometry analysis showed that the cells treated with either compound exhibited a significant decrease in the number of cells in the G2-M phase with respect to the control cells (*p* < 0.05) (Figure 3A,B, Table 1), indicating an S-phase cell cycle arrest. This effect corresponded with an increase in the number of cells arrested at the S-phase. Pterostilbene was significantly more potent than resveratrol in inhibiting cell cycle progression, showing effects at concentrations as low as 5 µM (*p* < 0.05) (Figure 3A,B, Table 1). At this concentration, pterostilbene had these percentages of cells in each phase: G1 = 53.4 ± 1.4, S = 34 ± 1.4, G2 =12.5 ± 0.2, while resveratrol had values of G1 = 64.8 ± 2.0, S = 16.3 ± 1.0, G2 = 18.3 ± 2.3. At a higher concentration (15 µM) both compounds significantly inhibited cells from entering into G2-M by arresting them in the S-phase, and difference between the extent of the arrest at this phase induced by the two compounds was significant (*p* < 0.05) (Figure 3A,B, Table 1). 

To confirm the cell cycle data, which indicated that both compounds are potent inhibitors of cells entering into G2-M, we investigated the status of the M-phase mitotic marker phospho-histone-H3 by immunocytochemistry (Figure 3C,D). At concentrations of 10 µM, both compounds significantly suppressed the amount of cells positive for the mitotic marker phospho-histone-H3, when compared to the untreated cells control. Although resveratrol significantly suppressed the abundance of phospho-histone-H3 (mitotic cells) at 5 µM, when compared to the control cells, pterostilbene at this concentration was significantly more potent than resveratrol (Figure 3D). Relative to the control, which was set at 100%, cells treated with 5 µM pterostilbene exhibited only 13.8% mitotic cells positive for the marker, which was significantly lower than the resveratrol-treated sample at this concentration, which had 60% mitotic cells (Figure 3D; *p* < 0.05). 

### 3.4. Downregulation of Viral Oncoprotein E6 and Upregulation of Active-Caspase-3 in HeLa Cells Treated with Pterostilbene and Resveratrol 

**Figure 4:**
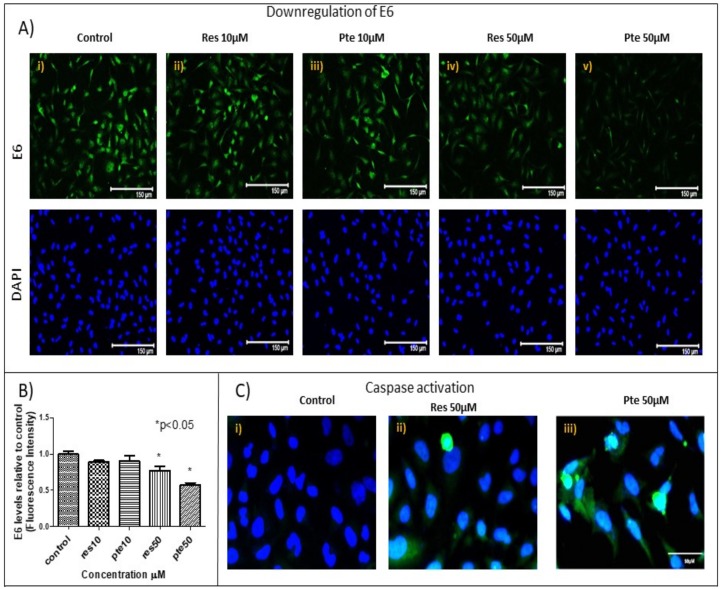
Downregulation of viral oncoprotein E6 and upregulation of active-caspase-3 in HeLa cells treated with resveratrol or pterostilbene: (**A**) HeLa cells immunostained for E6 levels (green) and counterstained with the nuclear dye 4’,6-diamidino-2-phenylindole (DAPI) (blue) after treatment with resveratrol (Res) and pterostilbene (Pte; 10 µM and 50 µM). Loss of E6 proteins are visually evident in cells treated with 50 µM of either resveratrol or pterostilbene. (**B**) Cell image analysis of the E6 fluorescent data revealed a significant 43% decrease of E6 protein levels in HeLa cells treated with pterostilbene at 50 µM and a 23% decrease of E6 levels in cells treated with resveratrol, both relative to the control. The graph represents data from experiments obtained from three independent experiments normalized to the control (mean % normalized to DAPI ± S.E.M.; * *p* < 0.05). (**C**) Immunofluorescent images probing for active-caspase-3 (green) shows a corresponding enhanced activation of this mediator of apoptosis by both resveratrol and pterostilbene.

In order to investigate how resveratrol and pterostilbene were affecting HeLa cell survival and cell cycle progression, we treated cells with either of the two compounds at sub-lethal (10 µM) and higher (50 µM) concentrations prior to analysis by immunostaining for E6, active caspase-3, and p53 (Figure 4A–C). At 10 µM, both resveratrol and pterostilbene failed to significantly affect levels of E6 and active caspase-3 levels relative to the control (Figure 4A,B). However, at 50 µM both compounds significantly suppressed E6 levels and elevated cleaved caspase-3 levels in treated cells relative to the untreated cells (Figure 3A–C). At this concentration (50 µM), pterostilbene was significantly more potent than resveratrol at suppressing E6 levels (resveratrol = 0.77 ± 0.11: 23% decrease vs. pterostilbene = 0.57 ± 0.06: 43% decrease; *p* < 0.05) and simultaneously elevating active caspase-3. It should be noted that we were unable to detect any noticeable differences in the sub-cellular localization of E6 in treated cells (Figure 4A). 

### 3.5. Upregulation of Tumor Suppressor Protein p53 in HeLa Cells Treated with Pterostilbene and Resveratrol 

**Figure 5:**
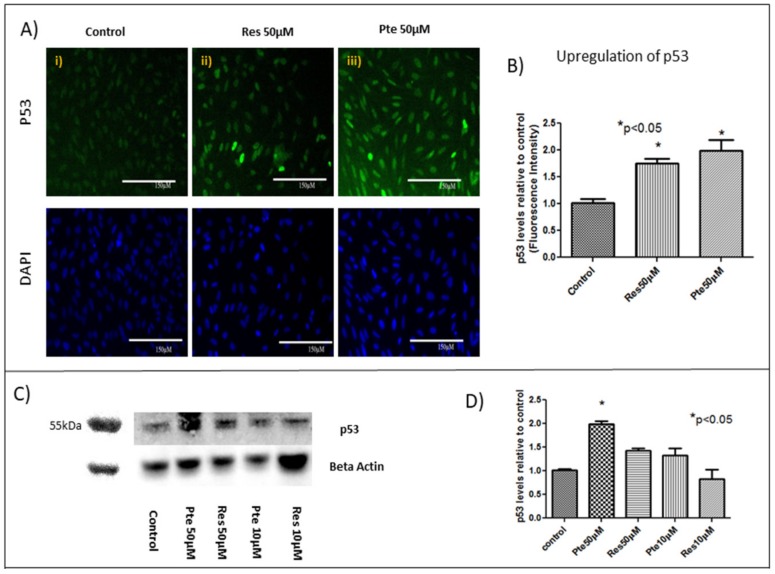
Upregulation of the tumor suppressor protein p53 in HeLa cells treated with resveratrol and pterostilbene: (**A**) Immunoflourescent images of p53 protein (green) untreated or after treatment with 50 µM of either resveratrol (Res) or pterostilbene (Pte) for 22 h. Levels of p53 are elevated in cells treated with either polyphenol. (**B**) Image analysis of p53 immunofluorescence indicates that pterostilbene treatment at 50 µM elicited a significant 2-fold increase in p53, while resveratrol exposure at similar concentrations induced a significant 1.75 increase in p53. The graph represents data from experiments obtained from three independent experiments normalized to the control (mean % normalized to DAPI ± S.E.M. * *p* < 0.05). (**C**) Western blot analysis also revealed that the elevation of p53 protein levels is evident in HeLa cells treated with 50 µM of resveratrol and pterostilbene; however, significant differences relative to the control were only reached with HeLa cells treated pterostilbene at 50 µM. (**D**) The graph represents data from experiments obtained from three independent experiments normalized to the control (mean % normalized to beta-actin ± S.E.M.; * *p* < 0.05).

Concomitant with E6 suppression, 50 µM pterostilbene treatment for 22 h caused an upregulation of p53 in HeLa cells (Figure 5A,B). When compared to the control, pterostilbene treatment elicited a 2-fold increase in p53 levels (staining normalized to DAPI; Figure 5B; *p* < 0.05). In comparison to the control, HeLa cells treated with 50 µM of resveratrol also caused an upregulation of p53 (1.75-fold increase; Figure 5A,B; *p* < 0.05) at 22 h. 

Total protein levels of p53 were also analyzed by Western blot in cells treated with either resveratrol (10 µM and 50 µM) or pterostilbene (10 µM and 50 µM) for 22 h (Figure 5C,D). Both compounds elevated p53 levels at 50 µM; however, significance was only noted in cells treated with pterostilbene at this concentration (Figure 5C,D). Although cells treated with pterostilbene at 10 µM tended to have elevated p53 protein levels relative to both the control cells and cells treated with 10 µM of resveratrol, these differences were not significant based on an ANOVA test (Figure 5C,D).

## 4. Discussion and Conclusions

In the current study, for the first time to our knowledge, we have compared the antitumor potency of resveratrol and pterostilbene on E6+ cervical cancer cells in vitro. We demonstrated that pterostilbene was significantly more potent than resveratrol in eliminating and in abrogating the clonogenicity of these cervical cancer cells (Figure 1). To assess and study the effects of the two compounds, we used a wide range of concentrations. Sub IC_50_ concentrations ranging from 5–20 µM were used to understand the action of these polyphenols at a low concentration. The results show that at these concentrations the polyphenols can inhibit cell division and migration. To further understand the cytotoxic mechanisms, it was imperative for us to look at supra IC_50_ concentrations. We used 50 µM to understand the mechanism of action. The clonogenic assay using this high concentration elucidates the long-term effect of these polyphenols on surviving cells even after the removal of treatment. While sub-IC_50_ values of both compounds inhibited the migration of E6+ cervical cancer cells, a higher sub-lethal concentration of resveratrol (20 µM) was needed to exert any significant inhibitory effect. Nonetheless, pterostilbene caused a more significant degree of inhibition to cell migration, attesting its superior antitumor potency (Figure 2). It is a notion held by cancer researchers that sub-IC_50_ concentrations of chemotherapeutic drugs are ineffective in curtailing tumor malignancy. However, surprisingly, our data shows that even at a low sub-lethal concentration (5 µM), pterostilbene is more effective than resveratrol as an antiproliferative agent against cervical cancer cells by triggering cell cycle arrest at the S-phase (Figure 3). In addition to being effective at sub-IC_50_ concentrations, the supra-IC_50_ concentration of pterostilbene (50 µM) was also superior to resveratrol (at 50 µM) in suppressing E6 while upregulating p53 and active-caspase-3 expression, thus causing a greater degree of apoptosis-mediated cell elimination. This observed suppression of E6 and upregulation of p53 is of paramount importance because HPV infection and cancer progression in cervical cells relies on the expression of the viral E6 oncoprotein which targets p53 for degradation by the ubiquitination [24,31]. Thus, untreated cervical cancer cells continue to proliferate in the absence of p53, unable to respond to cell stress and DNA damage. Our data indicates that resveratrol and pterostilbene may restore an adequate p53 response and ultimately act as anticancer plant compounds.

A comparative study between resveratrol and pterostilbene on colon cancer cells had shown pterostilbene to be a more potent anticancer agent compared to resveratrol [15]. Our first approach to understand the comparative efficacy of resveratrol and pterostilbene in HeLa cells was a cytotoxicity analysis, in addition to ascertaining the inhibitory concentration (IC_50_) (Figure 1 and Videos S1–S3). The results clearly indicated that pterostilbene could eliminate HeLa cells much faster and at a significantly lower concentration compared to resveratrol. We also further analyzed the cytotoxic potential of these polyphenols on a second cell line, E6-positive murine TC1 cells, and found a similar trend in IC_50_ results for resveratrol and pterostilbene, where pterostilbene is 2-fold more cytotoxic than resveratrol [32]. Since cancer cells are known to have enhanced clonogenecity [27,33], our study aimed to see the survival capability of the cells treated with supra-IC_50_ concentrations of either resveratrol or pterostilbene. Clonogenic studies show the long term-term effects of these polyphenols on cervical cancer cells after treatment for 24 h and then allowing the surviving cells to grow in normal growth medium for 15 days. Both compounds at supra-IC_50_ concentrations showed a dramatic decrease in the clonogenic capacity of the surviving cells. These results suggest that resveratrol and pterostilbene may suppress new tumor growth often seen in high-grade metastatic cervical cancer.

The migration of cancer cells is a very important factor responsible for the metastasizing of cancers [34]. Inhibition of migration can play a major role in checking the progression of cancer metastasis. Our study found that sub-cytotoxic doses of both compounds exhibit anti-migratory roles. These findings are supported by previous studies, which have shown that resveratrol shows anti-migratory activity by suppressing phorbol 12-myristate 13-acetate (PMA)-induced migration in cervical cancer cells [35]. Studies in hepatocellular carcinoma indicate that pterostilbene suppresses migration by downregulating MMP-9 expression [36]. These mechanisms might possibly be responsible for inhibiting migration in HeLa cells and remain to be determined in later studies. 

Previous cell cycle arrest studies of resveratrol on HeLa cells showed that all the cells were arrested at the S-phase and none remained in the G2/M-phase [37,38]. Pterostilbene shows cell cycle arrest in several cancer studies [39]; however, to the best of our knowledge, no such study on cervical cancer has been carried out. Our current study showed that pterostilbene shows markedly better efficacy than resveratrol in arresting the cell cycle at the S-phase. To further analyze the effects of the two compounds on cell cycle arrest, we looked at phospho-histone H3 as a marker for mitosis [40]. Our observations strengthen and confirm the results obtained from flow cytometric analysis indicating that although both compounds are able to arrest mitosis, pterostilbene has enhanced capacity to arrest cancer cell growth.

Although we initially used sub-lethal concentrations of the two compounds on HeLa cells to decipher their antitumor mechanisms in the context of cell cycle arrest, it was imperative for us to delineate the possible mechanism of elimination of HeLa cells by these compounds at higher concentrations. Pterostilbene is known to be effective on cervical cancer cells by Endoplasmic reticulum (ER)-mediated stress development as well as by targeting the Nrf-2 pathway [41]. In HPV+ cancer cells, the oncoprotein E6 degrades the tumor-suppressor protein p53 by targeting it for proteasomal ubiquitination, which has been shown to augment the tumorigenic characteristics of cancer cells [24,42]. In contrast, inhibition of E6 expression in the cancer cells would be expected to allow p53 protein to trigger apoptosis and cell cycle arrest. Our findings support this latter statement, with resveratrol and pterostilbene activating caspase-3 while simultaneously downregulating E6 and upregulating p53. Our findings are partly supported by previous studies indicating that resveratrol treatment on cervical cancer cell lines upregulates p53 [43]. Our findings are the first to show a direct upregulation of p53 in HeLa cells by another polyphenol, namely, pterostilbene. Importantly previous studies have shown that p53 and simultaneous caspase-3 activation might be the key for triggering apoptosis in HeLa cells [44]. Our experiments support this finding and ascertain that resveratrol and pterostilbene act as robust agents capable of regulating the p53-dependent apoptotic pathway. The p53 protein, which is usually very low in HeLa cells, was upregulated by resveratrol and pterostilbene, leading us to hypothesize that reactivation of p53 in treated HeLa cells is a possible mechanism of action of these compounds. 

Cervical cancer is a major concern in developing countries due to lack of affordable prophylaxis and treatment. As present modes of treatment like surgery, chemotherapy, or radiation involve high systemic toxicity, there is an urgent need to find affordable alternative therapies. Diet-based polyphenols like resveratrol and pterostilbene are therefore potential candidates for the effective therapy of cervical cancer with significantly low toxicity. We found pterostilbene to be a more potent anticancer agent than resveratrol in HeLa cells. This difference may be a function of pterostilbene being capable of upregulating p53 and downregulating E6 significantly more than resveratrol. As pterostilbene is non-toxic to normal cells [20], it has the potential to be a robust, cost-effective anti-E6+ tumor drug. Others have found that that pterostilbene possess greater bioavailability and stability [45] than resveratrol in vivo (80% vs. 20%)*.* Resveratrol has been shown to be non-toxic to several cells lines like glial cells and neurons, even after a treatment dose of 100 µM for 48 h [46]. Other studies on normal fibroblasts also state the non-toxicity of resveratrol at our observed potent anticancer concentrations [47]. Additionally, pterostilbene shows no toxicity at these concentrations in normal skin fibroblasts and myoblasts [48]. According to clinical studies, the safe dosage for resveratrol and pterostilbene is 5 g/day [49] and 250 mg/day [20], respectively. Our initial in vivo studies in the laboratory using a non-toxic dosage of both resveratrol and pterostilbene has shown promising results in inhibiting tumor growth in a model of cervical cancer [32]. Taken together, our findings support the further evaluation of pterostilbene as a possible therapy against cervical cancer.

Here, we show that pterostilbene potently suppresses HPV E6 expression (Figure 4) and efficiently eliminates HPV+ cells in culture by p53-mediated apoptosis (Figure 1 and Figure 5) while suppressing cell proliferation (Figure 3) and migration (Figure 2). We find that pterostilbene is a more promising agent against cervical cancer when compared to resveratrol. Based on such properties, the use of pterostilbene presents a relatively economical but highly hopeful therapeutic approach to treat HPV infections and cervical cancers. Our future studies will include signaling studies using HPV+ murine tumor models to confirm these observations in vivo.

## Figures and Tables

**Table 1:** Table showing the percentage of cells in each phase of the cell cycle (% ± S.E.M.) after treatment with different concentrations of resveratrol (Res) and pterostilbene (Pte).

	G1 ± S.E.M.	S ± S.E.M.	G2 ± S.E.M.
Control	64.1 ± 0.4	8.00 ± 2.5	27.7 ± 2.4
Res 5 µM	64.8 ± 2.0	16.3 ± 1.0	18.3 ± 2.3 ^^^
Pte 5 µM	53.4 ± 1.4 ^+^	34.0 ± 1.4 *	12.5 ± 0.2 ^^^
Res 10 µM	58.5 ± 0.2	26.5 ± 0.2 *	14.4 ± 1.0 ^^^
Pte 10 µM	54.3 ± 0.8 ^+^	35.6 ± 2.4 *	10.1 ± 1.5 ^^^
Res 15 µM	61.3 ± 1.9	27.1 ± 0.8 *^,#^	11.5 ± 1.2 ^^^
Pte 15 µM	52.3 ± 2.0 ^+^	40.1 ± 3.4 *^,#^	7.7 ± 0.5 ^^^

^+^ *p* < 0.05 relative to G1 control, * *p* < 0.05 relative to S control, ^^^ *p* < 0.05 relative to G2 control, ^#^ *p* < 0.05 relative to each other.

## Supplementary Materials

The following are available online at www.mdpi.com//2072-6643/10/02/243/s1, Video S1: Pterostilbene induces enhanced cell death compared to resveratrol: Time lapse video of cells untreated; Video S2: Pterostilbene induces enhanced cell death compared to resveratrol: Time lapse video of cells treated with 60 µM resveratrol; Video S3: Pterostilbene induces enhanced cell death compared to resveratrol: Time lapse video of cells treated with 60 µM pterostilbene obtained for 24 h showing elevated blebbing and cell death in pterostilbene treatment.
